# Orodental phenotype and genotype findings in all subtypes of hypophosphatasia

**DOI:** 10.1186/1750-1172-4-6

**Published:** 2009-02-21

**Authors:** Amélie Reibel, Marie-Cécile Manière, François Clauss, Dominique Droz, Yves Alembik, Etienne Mornet, Agnès Bloch-Zupan

**Affiliations:** 1 Department of Paediatric Dentistry, Faculty of Dentistry, University of Strasbourg, France; 2 Reference Centre for Oral Manifestations of Rare Diseases, Hôpitaux Universitaires de Strasbourg, Strasbourg, France; 3 Department of Paediatric Dentistry, Faculty of Dentistry, Nancy, France; 4 Department of Medical Genetics, Hôpitaux Universitaires de Strasbourg, Strasbourg, France; 5 SESEP Laboratory, Centre Hospitalier de Versailles, Le Chesnay, France; 6 EA2493 University of Versailles, Saint Quentin en Yvelines, Versailles, France; 7 IGBMC, Inserm, U964, CNRS, UMR7104, Illkirch, France

## Abstract

**Background:**

Hypophosphatasia (HP) is a rare inherited disorder characterized by a wide spectrum of defects in mineralized tissues and caused by deficiency in the tissue non-specific alkaline phosphatase gene (*ALPL*). The symptoms are highly variable in their clinical expression, and relate to numerous mutations in this gene. The first clinical sign of the disease is often a premature loss of deciduous teeth, mostly in the moderate forms.

**Aim:**

The purpose of this study was to document the oral features of HP patients and to relate theses features to the six recognized forms of HP in 5 patients with known genotype and to investigate the genotype-phenotype correlations.

**Methods:**

Clinical and radiographic examinations were carried out. We collected medical and dental history in the kindred and biochemical data. Finally, mutations in the *ALPL *gene were tested by DNA sequencing in SESEP laboratory.

**Results:**

We have for the first time related the known dental anomalies which occur as integral features of HP to the recognized clinical forms of HP. We also pointed out striking dental abnormalities which were never described in association with this rare disease. Accurate genotype-phenotype severity correlations were observed.

**Conclusion:**

This work allowed us to compare orodental manifestations in all the clinical forms of HP within the patient's sample. According to the severity of the disorder, some dental defects were infrequent, while other were always present. The long term prognosis of the permanent teeth varies from a patient to another. As premature loss of primary teeth is often the first, and sometimes the only visible symptom of the milder forms, the paediatric dentist plays a critical role in the detection and diagnosis of the disease.

## Background

Hypophosphatasia (HP) is an inherited disorder characterized by defective bone and tooth mineralization. HP is due to mutations in the liver/bone/kidney alkaline phosphatase gene (*ALPL*, MIM 171760) encoding the tissue-nonspecific alkaline phosphatase (TNAP) [1,2].

The disease is highly variable in its clinical expression, due to strong allelic heterogeneity in the *ALPL *gene. More than 190 mutations have been described and most of them (79%) are missense mutations. This diversity results in highly variable clinical expressivity and in a great number of compound heterozygous genotypes [3].

Clinical expression ranges from the extremes of stillbirth without mineralized bone to the isolated premature loss of primary teeth [2,4]. Six clinical forms of HP are currently recognized depending upon the age of onset: lethal perinatal (MIM 146300), prenatal (benign), infantile (MIM 241500), childhood (MIM 241510), adult and odontohypophosphatasia [5].

Severe forms of the disease (perinatal and infantile) are transmitted as an autosomal recessive trait, whereas both autosomal recessive and autosomal dominant transmission have been shown in moderate forms (childhood, adult and odontohypophosphatasia) [6]. Moreover, one case of *de novo *mutations was reported in 2005 [7].

The birth prevalence of severe HP has been estimated to be 1/100000 [4]. The incidence of moderate forms has never been estimated but is expected to be much higher [5].

In addition to clinical and radiographical examinations, laboratory assays are performed in case of suspicion of HP: total serum alkaline phosphatatase activity is markedly reduced. Molecular biology alone can establish the diagnosis. The detection rate is about 95% in the severe forms [5].

Currently, no established treatment exists for HP. Enzyme replacement using a substitutive enzyme targeting mineralized tissue seems to be a promising way of treatment which may become available in the next few years [8].

• In the lethal perinatal subtype, the patients show markedly impaired mineralization *in utero*. Some infants survive a few days but have respiratory complications due to hypoplastic lungs and rachitic deformities of the chest [5].

• In the prenatal benign form, despite prenatal symptoms, there is a spontaneous improvement of skeletal defect [5].

• Infantile HP may appear normal at birth; however, the clinical signs start during the first 6 months: hypercalcemia, rachitic deformities of the chest, premature craniosynostosis, widespread demineralization and rachitic changes in the metaphyses are classical features. In infants who survive (50%), there is often spontaneous improvement in mineralization and remission of clinical problems. Short stature in adulthood and premature shed of deciduous teeth are also common [1].

• The childhood form is heterogeneous. Clinical signs appear after 6 months of age. Skeletal deformities, a delay in walking, a short stature and a waddling are common features. Signs of intracranial hypertension or failure to thrive are typical. A history of fractures and bone pain usually exists as well. Premature loss of primary teeth is a trigger sign predicting the diagnosis [2]. Spontaneous remission of bone symptoms is well known, but the disease may re-appear in middle or late adulthood.

• The adult subtype develops during middle age. The first complaint may be foot pain, related to stress fractures of the metatarsals. Many of the patients present premature loss of permanent teeth [6].

• Odontohypophosphatasia seems to represent a form of the disease with only a dental phenotype. Indeed, this form is characterized by spontaneous exfoliation of fully rooted deciduous teeth, enlarged pulp chambers and root canals, not associated with abnormalities of the skeleton. Biochemical findings are generally indistinguishable from those of patients presenting mild forms of HP [5,9,10]. Odontohypophosphatasia should be considered in any patient with a history of early unexplained loss of teeth [1].

Oral findings may represent the main clinical manifestations in the moderate subtypes (childhood, adult and odontoHP), which are rarely diagnosed *per se*. Furthermore premature exfoliation of primary teeth is a "trigger sign" which can lead to a possible diagnosis. The most frequently reported oral symptoms are premature loss of anterior primary teeth without root resorption, reduced alveolar bone height. Hypoplasia of the cementum and enlargement of the pulp chambers and root canals are other findings [11]. Others features may exist, such as enamel hypoplasia, delayed dentine formation or delayed eruption [4,10,12-17].

The objectives of this study were to highlight dental aspects of HP in all clinical forms, including abnormalities of the permanent dentition and to relate the dental anomalies to the various subtypes of HP. Genotype-phenotype correlations were done in order to orientate the paediatrician and the paediatric dentist towards an earlier detection of the disease, thus leading to a better management of the patient.

## Methods

### Five patients were referred for oral and dental examination

#### Oral and radiographical examinations

Examination and dental care were carried out within the Paediatric Department of the Strasbourg Faculty of Dentistry and within the joint Reference Centre for Oral Manifestations of Rare Diseases, for the patients 1, 2, 3 and 5. Patient 4 was treated within the Paediatric Department of the Faculty of Dentistry of Nancy.

Periondontal examination consisted of the assessment of gingival inflammation, bleeding and probing depths. Estimation of the dental anomalies included abnormalities of number, size, shape and structure. We were also interested in disorders of eruption, impactions, the age at which teeth were lost and the exfoliation of posterior teeth.

On the radiographs (panoramic and peri-apical X-rays) we focussed on the thickness of the alveolar bone, the size of the pulp chambers and the degree of root resorption.

The orodental findings were documented using the Diagnosing Dental Defects Database record form D[4]. See  where a template of the form can be downloaded.

### Laboratory assays

The results of the biochemical investigations (serum AP value) were collated at the time of the diagnosis.

### Molecular biology

For all the patients, DNA sequencing of the *ALPL *gene was performed in the SESEP laboratory in Versailles (France). *ALPL *gene mutations database is available at: .

## Results

### Patients

#### Patient 1

The proband was 36 years of age at first examination. He is affected by the infantile form of HP. Despite the fact that the patient presented signs of HP from birth, the diagnosis was only establish at 2 years of age, following the loss of his anterior deciduous teeth. Molecular testing was done at 31 years of age. At birth, the patient was diagnosed with craniosynostosis, multiple fractures and had bone pain, which required several operative procedures. All primary teeth were lost by the age of 8 years, but his permanent dentition is still present at 36 years of age.

#### Patient 2

The boy was referred to the Paediatric Department of the Hôpitaux Universitaires de Strasbourg at 9 years of age. He has the infantile form of HP which was diagnosed at birth. He had experienced multiple fractures and craniosynostosis. Currently, he is confined to a wheelchair. Screening for the HP mutations was performed to confirm the diagnosis. At 9 years of age, he has already lost all mandibular primary teeth except the right lower first molar (84). The maxillary primary central incisors and second primary molars (51, 61, 55 and 65) had also been prematurely exfoliated. Currently, the patient is in the mixed dentition stage. The primary teeth still present are the maxillary lateral incisors, the maxillary canines, the maxillary first molars and the right lower first molar (52, 53, 54, 62, 63, 64 and 84), the permanent teeth present are the maxillary left central incisor, all four lower incisors and all first molars (21, 31, 32, 41, 42, 16, 26, 36 and 46).

#### Patient 3

A 10-months-old girl was referred by her paediatrician because of the spontaneous and unexplained loss of her primary lower central incisors (teeth 71 and 81), at 9 months of age. The patient's history reported normal growth. Except the absence of the central lower mandibular incisors, the oral examination was entirely normal, without inflammation of the gums. Thus, the child was referred to the geneticist for diagnosis of suspected HP.

Medical findings showed a slight delay in growth. Radiographs of the skeleton demonstrated general undermineralization of bones while biochemical investigations revealed a low level of serum AP and an urinary excretion of phosphoethanolamine, one of the substrates of the enzyme. Molecular analysis revealed 2 mutations on the *ALPL *gene and confirmed the diagnosis of a childhood subtype of HP. Today, at 4.5 years of age, the patient has lost all primary incisors, the lower primary canines and the upper right canine (51, 52, 53, 61, 62, 71, 72, 73, 81, 82 and 83). The lower first primary molars (74 and 84) are also very mobile. She also reports some difficulty in running and has bone pain.

#### Patient 4

The proband had her first visit at 1 year of age at the Paediatric Department of the University of Nancy Faculty of Dentistry. Her mother worried about the spontaneous loss of the lower right primary central incisor tooth (81). Although, the rest of the oral examination was normal, a diagnosis of HP was considered. At first, the laboratory assays did not show any abnormalities; the initial value of serum AP was normal (224 UI/L), and the diagnosis of HP was excluded. In the next two years, the patient lost 3 other primary teeth. The girl also complained of pain in her lower limbs.

New laboratory investigations were made and revealed a low value of serum AP (67 UI/L); normal values range for children from 123–283 UI/L. Molecular biology established the diagnosis of a childhood form of HP afterwards. At 4 years of age, she had already lost all her lower primary incisors (71, 72, 81 and 82).

#### Patient 5

A 30-months-old boy was referred by a paediatric dentist because of the premature loss of primary teeth. Except for eczema, the child presented with normal development. At 19 months of age, this child had spontaneously lost his four lower primary incisors (71, 72, 81 and 82) and the upper left primary incisors and canine (61, 62 and 63) following trauma. The oral examination did not reveal any gingivitis or bleeding. The patient was referred to the Department of Medical Genetics with suspected HP. Despite a normal growth pattern, radiographs of the skeleton revealed a distorted bone trabeculation with areas of decreased radiolucency and slightly deficient bone undermineralization. Biochemical data revealed a low level serum AP.

Molecular biology investigations showed one mutation on *ALPL *gene and confirmed the diagnosis of childhood HP. Today, at 6 years of age, the boy has lost all his mandibular and maxillary primary incisors and canines.

### Oral features of HP

The results for the dental features are summarized in Table 1.

**Table 1:** Dental phenotypes of 5 cases of HP

Dental features	1 infantile born in 1971	2 infantile born in 1998	3 childhood born in 2003	4 childhood born in 2004	5 childhoodHP born in 2002
Age at diagnosis	2 y	6 m	10 m	2,5 y	2,5 y
Age at examination	36 y	9 y	1 y	1 y	2,5 y
Loss of teeth < 1 year of age	+	+	+	-	-
Loss of posterior teeth	+	+	mobility	-	-
Inflammation of the gingiva Anomalies	-	-	-	-	-
number	-	-	-	-	-
shape: crowns	+	+	-	-	-
shape: roots and pulp chambers	+	+	+	+	+
size	-	-	-	-	-
structure	+	+	-	-	-
Impaction	-	+	+	-	-
Delay in eruption	+	+	+	-	-
Atrophy of alveolar bone	+	+	+	+	+
Type of dental phenotype	severe	severe	severe	moderate	moderate

Dental phenotype: a severe phenotype is associated with premature loss of teeth before 1 year of age and at least 3 types of dental anomalies. Moderate phenotype is associated with premature loss of teeth after 1 year of age and less than 3 types of dental anomalies.

Symbols: +: present, -: absent, m: months, y: year

**Figure 1:**
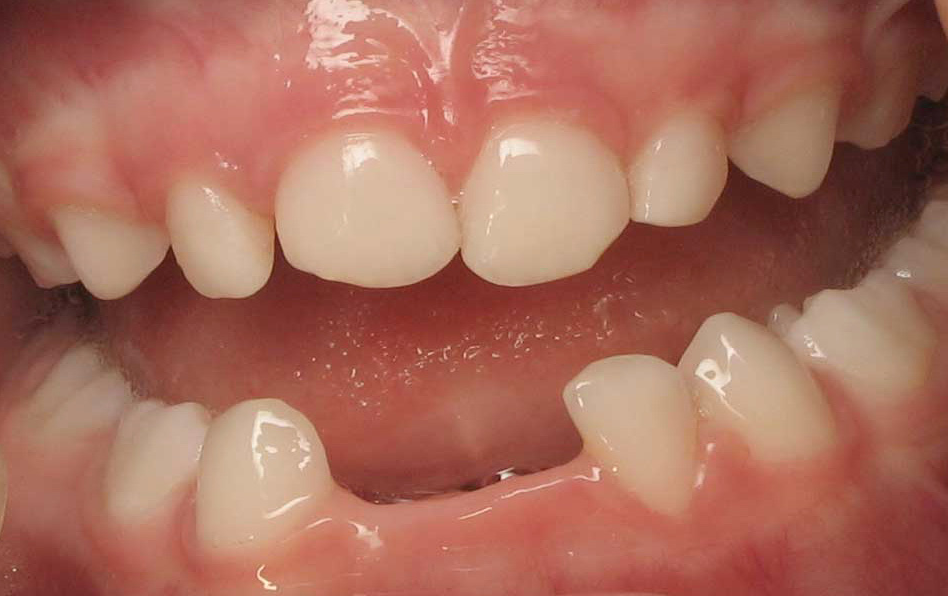
**Patient 4: Clinical view at 2.5 years of age**. The lower incisors (71, 72 and 81) are lost spontaneously without gingivitis.

**Figure 2:**
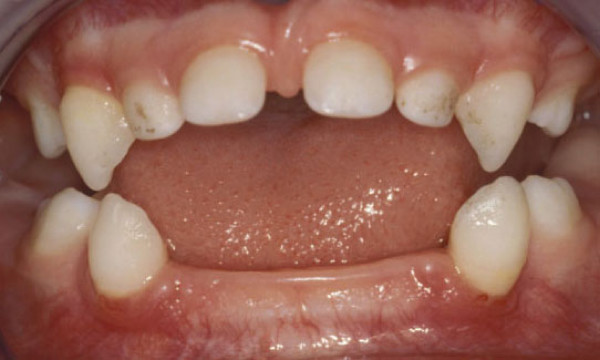
**Patient 3: Clinical view of the mandibular arch at 21 months of age**. There is no inflammation of the gingivae. The lower central incisors (71 and 81) have exfoliated at 9 months of age while the mandibular lateral incisors (72 and 82) exfoliated at 14 months of age.

All the patients had premature loss of their primary teeth, without gingival inflammation (Figure. 1, 2). The patients affected more severely (Patients 1, 2 and 3), lost teeth prior to one year of age and also exfoliated posterior teeth, while the patients midly affected (Patients 4 and 5) lost only their anterior teeth. Parents reported that before the loss of teeth, they had noticed that the teeth elongate, (like a continuing eruption). The history of premature exfoliation of teeth was only reported in the kindred of patient 2; his father losing his permanent teeth in his second decade, without signs of periodontitis. Unfortunately, most of the parents did not remember the age at which they lost their own primary dentitions.

**Figure 3:**
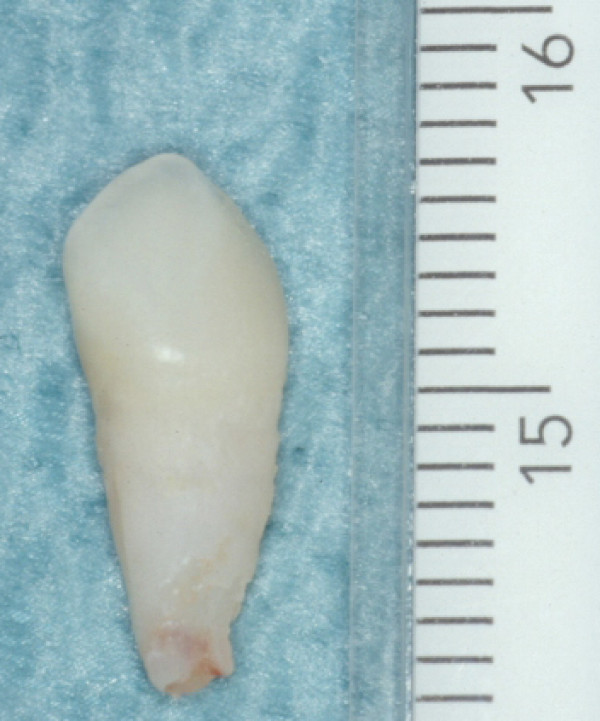
**Patient 5: Clinical view of the exfoliated mandibular right cuspid (tooth 83) at 4 years of age**. The root is intact.

During clinical examination we did not notice any bleeding, swelling or gingival recession; nor was there any periodontal pocketing. In all cases, the teeth were lost with intact and non-resorbed roots (Figure. 3).

#### Dental abnormalities

**Figure 4:**
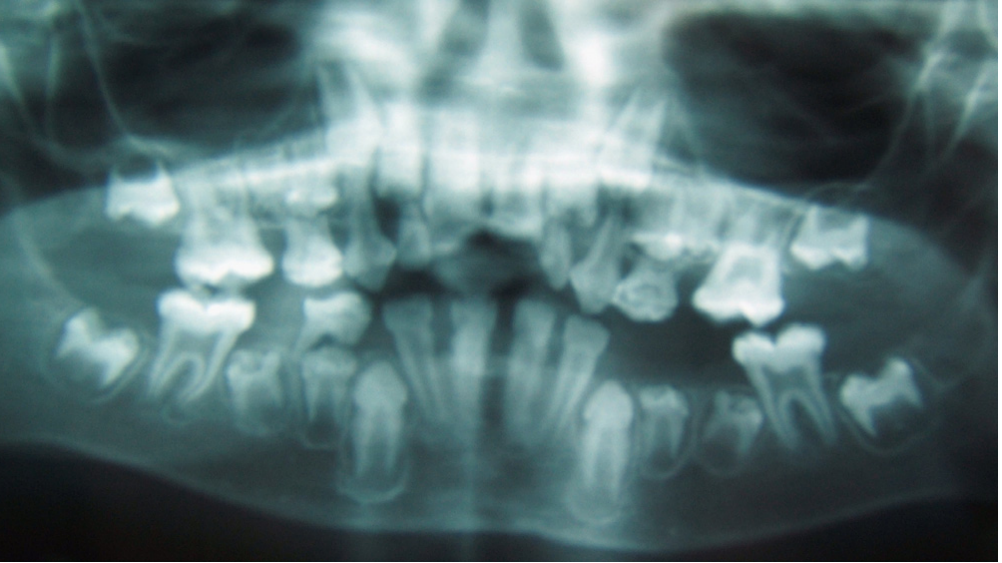
**Patient 2: Panoramic radiograph at 7.5 years of age**. The radiograph reveals enlarged pulp chambers and abnormalities of the shape of the crowns.

**Figure 5:**
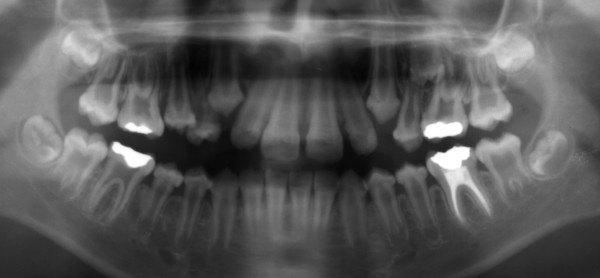
**Patient 1: Panoramic radiograph at 12 years of age**. The radiograph shows enlarged pulp chambers and abnormality of the shape of crown form. The second molars present more severe taurodontism than the first molars.

**Figure 6:**
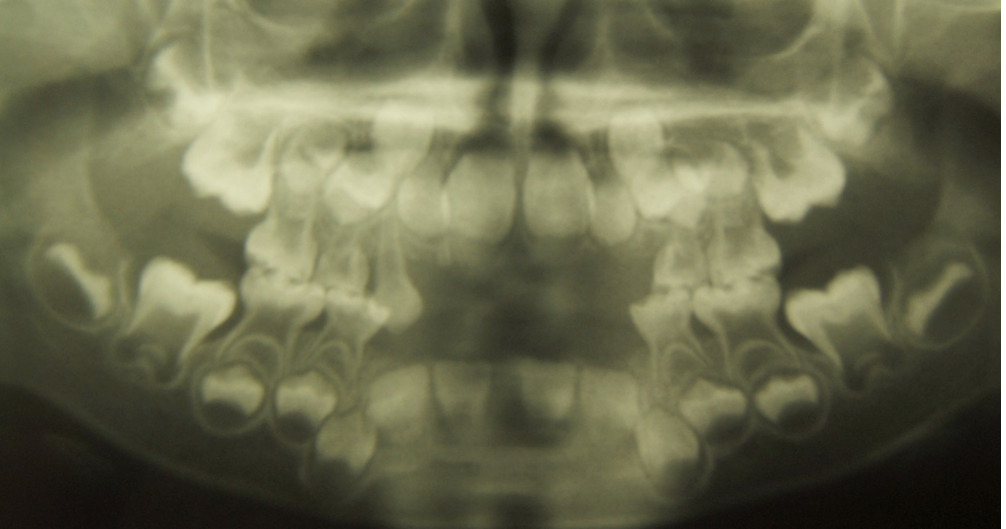
**Patient 5: Panoramic radiograph at 5 years of age**. The radiograph reveals enlarged pulp chambers and reduced thickness of both enamel and dentin.

**Figure 7:**
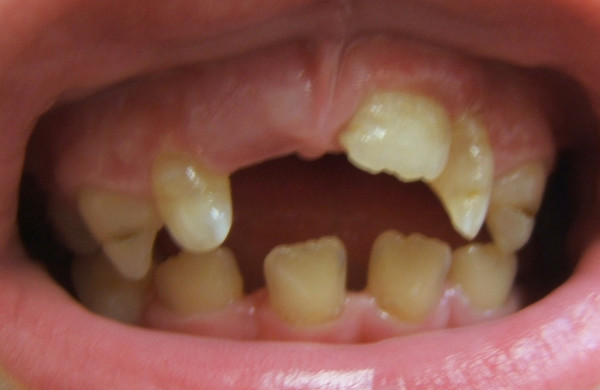
**Patient 2: Clinical view at 7.5 years of age**. The clinical examination reveals midcrown linear enamel hypoplasia of the maxillary canines (53 and 63) and delay in the eruption of the maxillary right central incisor (tooth 11) whereas the left maxillary permanent central incisor (tooth 21) had erupted at 6.5 years of age. The colour of the permanent teeth is dark yellow.

• Number: there were no missing or supernumerary teeth.

• Shape: abnormalities of the crown form of teeth were observed for the two infantile forms of HP. The crowns appeared small and bulbous, covered by thin enamel that did not appear to reach the cervical area. Cervical constriction was also present (Figure. 4, 5). Abnormalities of the shape of the roots and coronal pulp are a common features in all forms of HP. Primary teeth in all the patients presented enlarged pulp chambers and root canals with thin dentinal walls, visible both in anterior and posterior teeth (Figure. 4, 5, 6). This was considered to be true enlargement of the pulp chamber and not taurodontism as there was no apical displacement of the furcation in the molars, (the exception being in the case of patient 1 in whom the second permanent molars presented true taurodontism) (Figure. 5).

• Size: we did not observed microdontia or macrodontia.

• Structure: enamel hypoplasia was present in both patients affected by the infantile form of HP. Moreover, we noticed abnormalities of the colour of the permanent teeth in both patients being dark-yellow as observed in cases of dentin dysplasia (Figure. 7).

• Eruption: submerging impaction of both lower second primary molars (75 and 85) associated with intra-coronal pulp resorption was found in patient 3. Delay in eruption seems to be an important feature in patients 2 and 3 who presented this symptom.

• Horizontal alveolar bone loss of nearly half of the root length was seen on radiographs in patients 3 and 5.

Thus, classic features of HP; premature loss of primary teeth, reduced alveolar bone height and enlargement of coronal and root pulp chambers were found in all our patients. Moreover, in the severe forms of the disease, we observed other dental abnormalities such as changes in tooth shape, structure and eruption.

**Figure 8:**
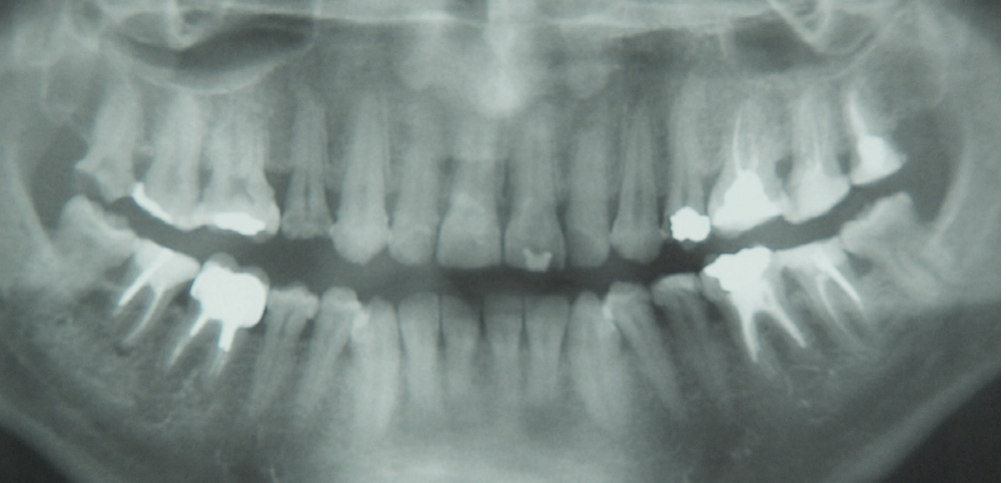
**Patient 1: Panoramic radiograph at 36 years of age**. The examination shows obliteration of the pulp chambers and reduced level of alveolar bone. The patient has a complete permanent dentition whereas he lost all this primary teeth prematurely.

Interestingly, we noticed an "improvement" or reduction in severity of the dental symptoms for the patient 1 between 12 and 36 years of age mainly though the apposition of dentin and the reduction of pulp spaces (Figure. 5 and 8).

### Genotype-phenotype correlations

We observed a good correlation of serum AP levels with the severity of the disease: the more severe the phenotype, the lower are the serum AP values (Table 2). This confirms that serum AP is a good marker of the severity of the disease [2].

**Table 2:** Genotype-phenotype correlations in HP patients.

	1 infantile	2 infantile	3 childhood	4 childhoodHP	5 childhoodHP
Dental phenotype	severe	severe	severe	moderate	moderate
AP values	12	35	44	67	88
Genotype	c.571>A (p.E191K) c.1361A>G (p.H454R)	c.571>A (p.E191K) c.550C>T (p.R184W)	c.526G>A (p.A176T) c.648+1G>A	c.1250A>G (p.N417S)	c. 212G>A (p.R71H)
Type of mutations	moderate and severe	moderate and severe	moderate and severe	severe	severe
Mode of inheritance	AR	AR	AR	AD	AD

Serum Alkaline Phosphatase (AP) values are those found at the age of diagnosis, i.e. between 6 months and 9 years of age. The normal values for children range from 123–283 UI/L.

Symbols: AR: autosomal recessive, AD: autosomal dominant

Footnote: Nucleotide and amino acid numbering are according to the international recommendation: the first nucleotide corresponds to the A of the ATG initiation codon.

The first amino acid corresponds to the ATG initiation codon.

The mutations detected by sequencing are reported in Table 2. In patients 1, 2 and 3, two heterozygous mutations were found, one of them corresponding to a severe mutation (c.1361 A>G and c.550C>T, respectively), the other being a mild mutation (c.571G>A) [18]. In patients 4 and 5, only one heterozygous mutation was detected, each of them was reported to have a severe effect [18]. These mutations were from paternal origin in each case. The father of patient 4 was asymptomatic but showed a low serum AP level (25 UI/L, normal range (35–90), suggesting dominant inheritance). No AP values were available for the father of patient 5.

Patients 1, 2 and 3 were affected by a severe dental phenotype while in contrast, patients 4 and 5 were much more midly affected.

Thus, the severe dental phenotype is correlated with a more severe general phenotype and the presence of 2 mutations on the *ALPL *gene.

In patients carrying only one mutation, althought this mutation is known to be severe, there is a moderately decreased serum AP value and a moderate form of the disease. This is due to the normal allele that provides enough AP activity to limit the dominant negative effect of the mutation.

## Discussion

For the first time, oral findings in five patients affected by several forms of HP are described in the same study. Our purpose was to highlight the differences between all the clinical forms. The classic features previously described in the literature were present in all the subtypes [10-13,19]. The time point of clinical manifestations is currently used to name the subtype of the disease, like perinatal, infantile (before 6 months) and childhood (after 6 months) forms. Indeed in our patients' sample such a classification is subject to questioning. For example even so affected by a clinically recognised severe infantile form, patient 1 was only diagnosed at 2 years of age. Patient 3 diagnosed at 10 months of age could be considered as a milder form of infantile with delay in diagnosis or as a childhood form. These considerations clearly show that especially the distinction of infantile and childhood hypophosphatasia forms is somewhat arbitrary and the phenotype is more a continuum than a precise subtype, even though, in the literature, these subtypes are usually cited.

Severe forms demonstrated premature loss of anterior teeth before one year of age and exfoliation of posterior teeth also. In the moderate forms, only primary anterior teeth exfoliated. Furthermore, others dental defects such as abnormalities of tooth shape, structure or eruption preferentially occured in the infantile form. We have shown that anomalies of the shape of the crowns and dentin defects, could also be a "trigger feature" pointing to a diagnosis of HP in the severe forms. In addition, we reported bilateral impaction associated with intra-coronal pulp resorption in HP a feature never described in HP before. Nevertheless, the submerged impacted second primary molars can also be seen in the normal child population where permanent second premolars are congenitally absent.

We did not find any patient with inflammation of the gingiva or periodontitis. The presence of periodontitis in HP is controversial in the literature: Baab *et al*., Plagmann *et al*., and Watanabe *et al*. described an association between HP and periodontitis [16,20,21], while Valenza *et al*. found no signicative differences in subgingival microbiota of healthy patients and patients suffering from HP [22]. Baab *et al*. and El-Labban *et al*. discussed the possibility that the weakened periodontal ligament was considerably more susceptible to bacterial invasion and could promote periodontitis [20,23]. Attachment loss in HP may be the result of a cemental or alveolar bone defects rather than the outcome of inflammation induced by bacterial growth. Longitudinal studies will be of interest to follow changes of the microbiota over time [22].

The eruption of permanent teeth must be considered carefully. As in patient 1, some patients could have lost all their primary teeth prematurely and later present in adulthood with a complete permanent dentition and few or no dental defects [14]. This may be attributable to a progressive general improvement of affected patients from infancy to adulthood [6]. Lepe *et al*. described a case of childhood HP without dental manifestations in adulthood [14]. This hypothesis could explain, in addition to incomplete penetrance of the disease, the reason why most of the parents have no detectable dental defects in their permanent dentition. Some may however have a history of early loss of primary teeth and this should be investigated during the medical and familial history. A precise dental status of the family would indeed be also very instructive especially in the cases of compound heterozygosity where parents are affected by one mutation. In another cohort [9] especially these parents were also shown to have clinical features of dental disease. In our group only the father of patient 2 had a history of early loss of primary teeth.

Moreover, there is no previous case report in the literature which describes patients with more defects in the permanent than in the primary dentition. Another point to be considered is that: at the time of the occurrence of the disease in the adult form, odontogenesis has already been completed. Thus, premature loss of teeth alone should be looked for in patients affected by the adult form without the presence of other dental anomalies. Nevertheless, if some dental anomalies are present, it could be possible that the patient has actually a childhood subtype undiagnosed at the time.

Of interest, are the strong differences between the panoramic views at 12 and 36 years of age for the patient 1. At 13 years old, the patient presented with enlargement of the pulp chambers, a slight reduced alveolar bone height and small bulbous crowns, covered by thin enamel that does not seem to reach the cervical area. Twenty years later, all the teeth are still present, the level of the alveolar bone is almost the same the pulp chambers have secondary dentine apposition. Enamel hypoplasia and a reduction of the enamel thickness were noted in the initial radiograph. A hypothesis of a delay in dentin formation could explain these changes.

In some reported cases, the same defects were observed in both dentitions [13,15,23] and in other cases the permanent dentition appears healthy [14]. Thus, we can not establish a prognosis for the permanent dentition in the light of the changes in the primary one. This indicates, that carefull monitoring is necessary. Suitable prophylactic programs should be launched, aiming at preserving general health and a healthy oral cavity avoiding any bacterial invasion which could trigger periodontal disease [11].

Alkaline phosphatase (AP) participates in tooth formation and is seen in dental and peridental cells – ameloblasts, odontoblasts, cementoblasts and osteoclasts [24]. Moreover, the enzyme is also expressed very intensively in "satellite" cells: osteoprogenitor, supra-ameloblastic, and subodontoblastic cells. This enzyme plays a significant role during two critical phases, initiation and completion of dental biomineralization. AP is intensely expressed during terminal differentiation of osteoblasts and in initial biomineralization and decreases when bone mineralization has been achieved [24]. These findings and the role of the enzyme during these two essential phases could explain why different types of dental anomalies could be present in HP patients. Van den Bos *et al*., observed that both acellular and cellular cementum formation was affected. They also showed that mineralization of dentin is less likely to be under the influence of the inhibitory action of inorganic pyrophosphates (one of the substrates of TNAP) than mineralization of cementum. This may suggest that different regulatory mechanisms operate for mineralization in these two tissues [25].

More than 190 mutations are currently described worldwide in HP patients. This diversity of mutations results in variable clinical expressions even among the severe or moderate types [9]. This may explain the heterogeneity of the phenotype and the overlap of the clinical subtypes: for instance, infantile and childhood HP share some clinical symptoms, and patients with adult HP often recall childhood rickets or premature exfoliation of primary teeth [5,15,26]. Depending upon the impact of the mutation on enzyme function, the clinical manifestations are highly variable, which is reflected by the age of onset [27].

Here patients 1, 2 and 3 carry two heterozygous mutations, one moderate (c.571G>A or c.526G>A), the other one severe (c.1361A>G, c.550C>T or c.648+1G>A) [18]. This is a common situation in non-lethal forms of HP, the moderate allele allowing the production of AP activity from which depends the severity of the disease. The most midly affected patients of our series were found heterozygotes for the missense mutation c.1250A>G and c.212G>A. These mutations have a dominant negative effect that could explain the mild expression of the disease at the heterozygous state. Indeed, by site-directed mutagenesis we found that cells transfected with the mutations R71H and N417S, and co-transfected with the wild type allele, exhibit 30.5% and 26.5% of wild type alkaline phosphatase activity, respectively, [Fauvert *et al*. manuscript in preparation].

We found in our series very good correlation between the severity of the symptoms and the AP value at time of diagnosis. Variation in clinical expression is currently known to correlate well with variable residual enzyme activity. Generally, the more severe the disease, the lower the serum AP activity level appropriate for age [2]. Nevertheless, AP activity varies greatly with age and sex [5]. Patient 4, at 1 year of age had a normal serum AP value which at first excluded the diagnosis of HP, but when tested again 2 years later he had a decreased value. Thus, molecular biology is crucial to establish the definitive diagnosis when biochemical and clinical data are not clear enough [5].

Screening for the mutations in the *ALPL *gene is also of greater importance for prediction of recurrence risk in future pregnancies, carrier detection and prenatal diagnosis. The molecular diagnosis of each patient increases the knowledge of the level of severity of symptoms associated with each *ALPL *mutation and improves our ability to provide a better prognosis.

According to Hartsfield, premature exfoliation of primary teeth in children younger than 5 years of age should suggest a genetic or a systemic disease in the absence of trauma, especially, in children younger than 3 years of age [26]. The differential diagnosis includes inherited immune diseases (Papillon-Lefèvre syndrome, Chediak-Higashi syndrome, neutropenias), inherited non-immune diseases (Ehlers-Danlos syndrome, Coffin-Lowry syndrome, hypophosphatemic rickets and dentinal dysplasia type I), sensory neuropathies neoplasias and early-onset periodontitis [26]. In moderate HP, the premature and spontaneous loss of primary teeth is in most the cases the only symptom. There is no pain and no association with others oral features as inflammation of the gums, ulcerations or abscesses [13,21]. Thus, this symptom is a trigger sign in the milder forms of HP pointing to or predicting the diagnosis [16,26]. Premature and spontaneous loss of teeth alone without any history of trauma, should lead to further investigation. For any patient with this symptom, the diagnosis of HP should be considered.

## Conclusion

Milder forms of HP still pose problems for timely and accurate diagnosis [28,29]. Here, we have for the first time related the known dental anomalies which occur in HP to the various subtypes of HP. The paediatric dentist plays a crucial role in the early detection of HP, as he is, the most likely, the first person to meet the patient [27]. Such was the case for our mildly affected patients and shows the importance of accurate dental assessment and analysis in pointing towards a diagnosis. An early correct diagnosis, besides defining the disease, enables the provision of effective therapy, when such becomes available in the future.

## Abbreviations

HP: hypophosphatasia; *ALPL*: tissue non-specific alkaline phosphatase gene; DNA: desoxyribo nucleic acid; TNAP: tissue-nonspecific alkaline phosphatase; AP: alkaline phosphatase.

## Consent

Written consent for publication was obtained from the patients and/or their relatives.

## Competing interests

The authors declare that they have no competing interests.
